# A Comparative Analysis of Prenatal Care and Fetal Growth in Eight South American Countries

**DOI:** 10.1371/journal.pone.0091292

**Published:** 2014-03-13

**Authors:** Cristina Woodhouse, Jorge Lopez Camelo, George L. Wehby

**Affiliations:** 1 College of Medicine, University of Iowa, Iowa City, Iowa, United States of America; 2 Centro de Educación Médica e Investigación Clínica (CEMIC); Consejo Nacional de Investigaciones, Científicas y Tecnológicas (CONICET), Buenos Aires, Argentina; 3 Associate Professor, University of Iowa, Research Associate, National Bureau of Economic Research, Dept. of Health Management and Policy, College of Public Health, University of Iowa, Iowa City, Iowa, United States of America; University of Alabama at Birmingham, United States of America

## Abstract

There has been little work that comprehensively compared the relationship between prenatal care and infant health across multiple countries using similar data sources and analytical models. Such comparative analyses are useful for understanding the background of differences in infant health between populations. We evaluated the association between prenatal care visits and fetal growth measured by birth weight (BW) in grams or low birth weight (<2500 grams; LBW) adjusted for gestational age in eight South American countries using similarly collected data across countries and the same analytical models. OLS and logistic regressions were estimated adjusting for a large set of relevant infant, maternal, and household characteristics and birth year and hospital fixed effects. Birth data were acquired from 140 hospitals that are part of the Latin American Collaborative Study of Congenital Malformations (ECLAMC) network. The analytical sample included 56,014 live-born infants (∼69% of total sample) with complete data born without congenital anomalies in the years 1996–2011 in Brazil, Argentina, Chile, Venezuela, Ecuador, Colombia, Bolivia, and Uruguay. Prenatal care visits were significantly (at p<.05) and positively associated with BW and negatively associated with LBW for all countries. The OLS coefficients ranged from 9 grams per visit in Bolivia to 36 grams in Uruguay. The association with LBW was strongest for Chile (OR = 0.87 per visit) and lowest for Argentina and Venezuela (OR = 0.95). The association decreased in the recent decade compared to earlier years. Our findings suggest that estimates of association between prenatal care and fetal growth are population-specific and may not be generalizable to other populations. Furthermore, as one of the indicators for a country’s healthcare system for maternal and child health, prenatal care is a highly variable indicator between countries in South America.

## Introduction

Prenatal care is arguably one of the most accessible interventions for maternal health and fetal development. Lack of and inadequate prenatal care have been associated with major poor fetal/infant health conditions including low birth weight (LBW)[1]–[4], preterm birth (PTB), and neonatal and infant mortality [1], [2], [5]. LBW affects about 20 million infants each year or close to 15.5% of live births worldwide and is a contributing factor for 60–80% of neonatal deaths [6]. Estimates suggest a 25 times greater risk of death for newborns weighing less than 2500 g and a 100 times greater risk for newborns weighing less than 1500 g [7]. In addition to its link to mortality, LBW is associated with child morbidity, developmental disabilities, and adverse consequences to health and wellbeing in adulthood [8], [9]. LBW has been associated with coronary disease, hypertension, stroke, diabetes, obesity, a variety of other medical conditions, and lower socioeconomic achievements in adulthood.

Close to 96.5% of the world’s LBW infants are born in less developed countries [6], making these an important setting for studying prenatal care, a relatively low-cost health intervention that can be made accessible to the majority of the population. Only one third of pregnant women in low-income countries obtain adequate prenatal care (at least 4 visits) [10]. Therefore, there is still a great opportunity to expand access to prenatal care for most pregnant women in less developed countries. However, most research has focused on developed countries creating a significant need for studying developing nations. In this regard, South America is a continent of interest. Neonatal mortality is more than twice as high in Latin America compared to developed regions (10 versus 4 neonatal deaths per 1,000 in 2011) [11]. South America’s average LBW rate is 9.6% which falls between the average rates for developed and other less developed countries. However, the total number of LBW infants in South America is nearly double that in all of North America [12]. Furthermore, there is an interesting variation in LBW prevalence between South American countries; for example rates in 2005–2010 were as low as 6% in Bolivia and Chile but 9% in Uruguay [10]. Similarly, neonatal mortality varied from 5 per 1,000 in Chile to 23 per 1,000 in Bolivia in 2010 [10]. This variation suggests a rather dynamic etiology of LBW and other major infant health indicators and provides a valuable opportunity for cross-country comparisons.

Previous studies using South American data have mostly focused on single countries and used different samples and analytical models, making it hard to comprehensively compare the relationship between prenatal care and fetal growth across multiple countries. The literature suggests that prenatal care is positively associated with birth weight in several South American populations such as in Brazil [3], [4], [13], Argentina [14], [15], Chile [16], and Uruguay [17]. However, the results from single-country studies may not be generalizable to other countries since differences in population demographics, economics, health, and healthcare systems may modify the association between prenatal care and fetal growth across countries. South American countries vary extensively on several of these characteristics. For example, GDP per capita in 2010 varied from $1,979 in Bolivia to $12,640 in Chile [18]. Maternal factors vary as well with adolescent fertility rates ranging from 55per 1,000 females age 15–19 in Argentina to 88 per 1,000 in Venezuela and female literacy rates ranging from 87% in Bolivia to 98% in Uruguay [19]. Therefore, country comparisons are useful for understanding the background of differences in infant health between populations. What is needed is a study that analyzes similarly data collected across multiple countries using the same analytical approach. Indeed, evaluating this generalizability is an important empirical question. What is needed is a study that analyzes similarly data collected across multiple countries using the same analytical approach.

To our knowledge, very few studies have done such a comparative analysis. One relatively old study of prenatal care and LBW used an international data collection program for 6 countries from 4 continents [16]. The study found a positive association overall but varying estimates between countries. Another study combined 4 South American countries (Brazil, Bolivia, Colombia, and Peru) in one regression and included interaction terms between the country indicators and prenatal care measures, which were significant, indicating different associations between countries [20]. However, the sample size was limited and the analysis assumed that other covariates had similar associations with BW across these countries, which is a strong assumption.

We evaluate the association between prenatal care and fetal growth – birth weight adjusted for gestational age – using the same analytical model and similarly enrolled samples and collected data from eight South American countries including Brazil, Argentina, Chile, Venezuela, Ecuador, Colombia, Bolivia, and Uruguay; the last five of these countries have had very little representation in this literature. To the best of our knowledge, no previous study has conducted such comparative analysis using the same data source, methodology, and measures for these countries.

## Methods

The study was approved by the University of Iowa Institutional Review Board and by the Ethics Committee of the Centro de Educación Médica e Investigación Clínica (CEMIC) in Argentina.

### Data/Study Sample

The data for this study were from the Latin-American Collaborative Study of Congenital Malformations (ECLAMC). ECLAMC is a program for epidemiological surveillance and investigation of congenital anomalies that began in Argentina in 1967 and has since been extended to hospitals in 11 other Latin American countries [21]. ECLAMC is built on a voluntary participation model under which affiliated hospitals and physicians (mostly pediatricians) identify infants born with anomalies in the affiliated hospitals as well as infants without birth defects who are matched one-to-one to the affected infants by date and hospital of birth and sex [21]. The affiliated physicians collect data on prenatal factors, maternal health, socioeconomic and demographic characteristics, and infant health indicators including birth weight (BW) from interviews with the mothers before discharge and from abstracting hospital records [21]. The same questionnaire and data collection protocols are used by the physicians across all hospitals and countries [21]. Parents of children enrolled in ECLAMC completed consent forms for enrolling into ECLAMC and using the data in research projects. ECLAMC-affiliated physicians receive standard training before beginning data collection and attend annual meetings during which refresher training is provided as needed.

Our sample for this study included live-born infants *without* birth defects enrolled into ECLAMC and excluded infants born with birth defects as these may modify the relationship between prenatal care and BW [22] and may affect BW [23]. ECLAMC focuses primarily on enrolling live-born infants and does not collect data on miscarriages; only few stillbirths were enrolled during the study years. We studied eight countries: Brazil, Argentina, Chile, Venezuela, Ecuador, Colombia, Bolivia and Uruguay, which have the largest sample sizes in ECLAMC. Other countries represented in ECLAMC such as Paraguay and Peru have much smaller samples given their coverage and participation in ECLAMC and were therefore not analyzed. The number of ECLAMC-affiliated hospitals within each country sample was as follows: 30 in Brazil, 54 in Argentina; 15 in Chile, 4 in Venezuela, 12 in Ecuador, 11 in Colombia, 5 in Bolivia, and 9 in Uruguay.

ECLAMC began collecting data on prenatal care use after 1995. Therefore, our data sample included infants born between 1996 and 2011, which is the most recent year with available data. Ecuador and Colombia began data collection in ECLAMC in 2001 and provided data for 2001–2011. The sample from Uruguay included births from 1996 to 2008. Some exclusion criteria were used to avoid data recording errors. Infants born weighing less than 500 g or more than 6,000 g were excluded consistent with most previous studies on prenatal care and BW. Similarly, the mothers in our study were limited to those between 13 and 49 years of age. Finally, only infants with complete data on all model variables were included with an exception for paternal education and employment; missing data on these two control variables were represented by indicators to avoid further sample size reduction.

Prenatal care was measured by number of prenatal care visits. We chose this measure in order to examine the average association per prenatal visit and because it is one of the most commonly used measures in studies of prenatal care [13]–[15]. Unlike prenatal care adequacy measures that assume some (albeit undefined) scale of prenatal care effectiveness by considering the number of prenatal visits to have been adequate or not based on an arbitrary algorithm that accounts for delay in initiating prenatal care and gestational age at birth, this generic measure is more appropriate by allowing for estimating the average association of an additional prenatal visit [22]. We do not use delay in initiating prenatal care because previous work has shown a strong self-selection bias in this measure due to unobservable confounders, in that women at greater risk of having a lower BW child initiated prenatal care earlier, which biased the association with BW substantially downward (towards no-association) [14].

In the years 1996–2003 and in 2005, visits greater than 9 were capped at 9 visits during data entry into the ECLAMC database (this occurred in 1996–2002 in the study countries and in 2005 in Venezuela). This may bias the average association per visit upward since multiple visits (in cases where total visits exceeded 9) may be combined in the ninth visit. Therefore, in addition to analyzing the main sample from each country including mothers who had 9 or more visits and in order to check the sensitivity of our results to this data limitation, we re-analyzed a subsample that excluded these observations. In other words, we only included in this additional analysis women with a range of prenatal visits from 0 to 8 and omitted those that had 9 or more visits. The majority of women (from 54% in Uruguay and 73% in Brazil to 96% in Bolivia) who obtained prenatal care had 8 or fewer visits except for Chile (40%). Therefore, the results from this additional analysis would be applicable to the majority of women who sought prenatal care in most study countries consistent with most prenatal care guidelines for low-risk pregnancies which typically focus on fewer than 9 visits such as in Brazil where a minimum of 6 visits are recommended [24].

We measured fetal growth by BW, adjusted for gestational age. We used two measures of BW. The first was a continuous measure in grams in order to utilize all available variation and derive an estimate of the average association of BW in grams per prenatal visit. The second was a binary indicator for LBW (<2500 grams) given its strong correlation with child survival and development as well as long term outcomes.

### Analysis

We evaluated the association between prenatal care and fetal growth using regression analysis separately for each country. Specifically, continuous BW and LBW were modeled as a function of number of prenatal care visits and adjusting for gestational age and several other conceptually and empirically relevant infant, maternal, and household characteristics that may relate to both prenatal care and BW as reported in several previous studies (25, 27). We controlled for gestational age since longer pregnancies may have more visits but also greater BW. With this adjustment, the variation in BW represent variation in fetal growth rate since gestational length is held constant and the estimates for prenatal care are interpreted as representing association with fetal growth rate. The other covariates were infant sex and racial ancestry (Native, African, or other), maternal age (divided into four categories: 13–19, 20–25, 26–34 and 35–49 years), length of time of cohabitation of parents prior to infant birth (used as a proxy for marital status which is not measured in this dataset), maternal health and fertility history (vaginal bleeding during first trimester, presence of acute illness during pregnancy, presence of chronic illness during pregnancy, number of previous live births, number of previous spontaneous abortions or stillbirths, and history of difficulty conceiving), all of which could influence self-selection into prenatal care but also modify BW and LBW risk as shown in several previous studies including those utilizing the same data source as this study(25, 27). Similarly, we controlled for several household enabling characteristics related to socioeconomic status including maternal education level, maternal employment, paternal education level, and paternal employment; separate dummy variables representing observations with missing data on the paternal variables were included. The model also included dummy variables for year of infant birth in order to account for time trends in BW and prenatal care use, and dummy variables for the hospital of birth to account for geographic variation in prenatal care and BW and for self-selection into hospital of birth based on unobservables related to both prenatal care use and fetal growth. The study variables were systematically measured over the study period so there is no concern about bias due to changes in measurement techniques. However the year fixed effects would capture such biases if they existed in the data.

We directly controlled for these covariates in order to account for all the confounders measured in this dataset. All of the covariates adjusted for in the analysis are unlikely to be on the causal pathway between prenatal care and BW but rather typical confounders associated with both BW and frequency of prenatal care visits as shown in previous studies. The main exception is gestational age which can be affected by prenatal care, but is also a strong confounder in that prenatal care visits increase with longer gestation; therefore, excluding gestational age may seriously bias the association between prenatal visits and BW. All the demographic, socioeconomic, and fertility history variables controlled for are not on the causal pathway between prenatal care and BW and precede the current pregnancy included in the analysis. Similarly, the three variables for maternal health during pregnancy that we adjusted for (acute illnesses, chronic illnesses, and vaginal bleeding in the first trimester) are also relevant for self-selection into prenatal care and frequency of visits. It is possible that maternal health is influenced by prenatal care use, but it is unlikely that the presence of chronic conditions during pregnancy is impacted by prenatal care. Similarly, occurrence of acute illnesses is largely unrelated to prenatal care, as is the occurrence of vaginal bleeding early in the pregnancy. While these three variables may be slightly influenced by prenatal care, they are related to BW through other pathways and the threat of excluding them given their impact on self-selection into prenatal care likely exceeds the gain from omitting them because they are marginally influenced by prenatal care.

The model was estimated using ordinary least squares (OLS) for continuous BW and by logistic regression for LBW. Standard errors were clustered at the hospital of birth using a Huber-type variance-covariance estimator [25]. Given the potential self-selection into prenatal care, we recognize that we can only estimate the association between prenatal care and BW in this study.

We first estimated these models over the entire observed period for each country. Next for the five countries with data on the entire period from 1996 to 2011 (Brazil, Argentina, Chile, Venezuela, and Bolivia), we re-estimated the models by stratifying the samples into two birth periods –1996–2002 and 2003–2011– in order to evaluate time trends in the association between prenatal care and fetal growth. Ecuador and Colombia were excluded from this stratified analysis since they had no infants born prior to 2001 in the sample. Uruguay was also excluded from this analysis because around 80% of the sample was born before 2003 and its sample only includes births through 2008 as mentioned above. The birth year cutoff of 2003 was chosen to balance the size of the stratified subsamples as much as possible between the two periods. In order to avoid a bias from recording the 10 or more prenatal visits at 9 visits during data entry in earlier years as described above, we limit this stratified analysis to mothers who used 8 or fewer prenatal visits (including those who did not use prenatal care).

## Results

### Sample Description

**Figure 1:**
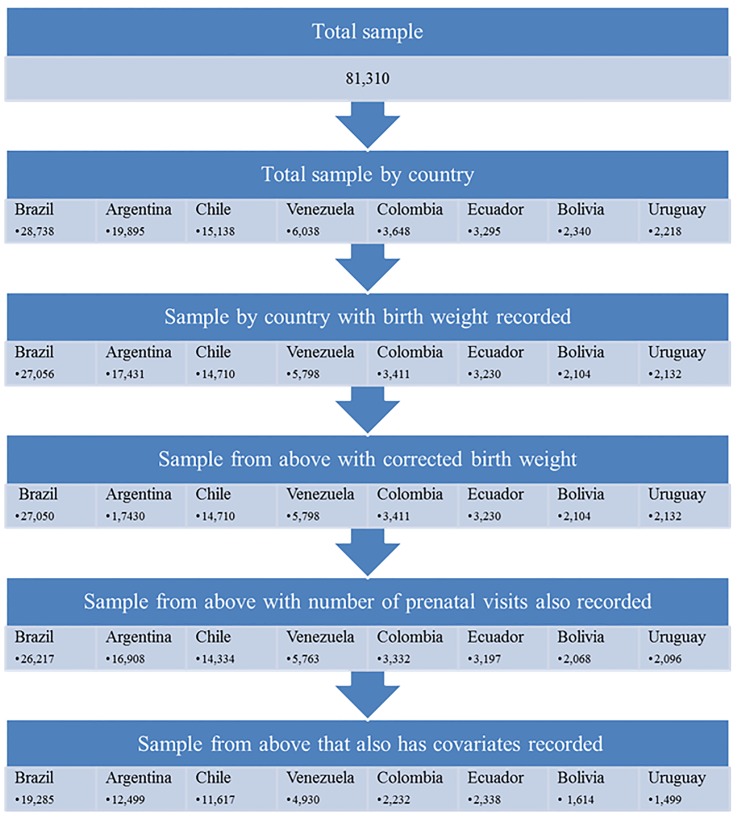
Sample construction chart.

The original sample included 81,310 infants from the study countries born between 1996 and 2011. After the study exclusions described above, the analytical sample included 56,104 infants distributed by country as follows: 19,285 in Brazil, 12,499 in Argentina, 11,617 in Chile, 4,930 in Venezuela, 2,232 in Colombia, 2,338 in Ecuador, 1,614 in Bolivia, and 1,499 in Uruguay. Figure 1 shows a detailed description of sample construction and exclusions by country. Online supplementary Table S1 shows the frequency of complete data for each study variable in the sample with complete data on prenatal care and BW. Gestational age had the highest rate of missing data compared to other covariates except in Chile and Bolivia.

Descriptive summaries of the study variables for each country sample are shown in Table 1. Mean number of prenatal care visits ranged from 4.5 (Bolivia) to 8.2 (Chile). Mean BW ranged from 2991 (Colombia) to 3375 grams (Chile). The LBW rate ranged from 5.09% in Chile to 14.07% in Colombia. The most common maternal age group was 20–25 years for all countries (34–38%) except Chile, Colombia, and Uruguay which had the most mothers between 26 and 34. About 24% (Bolivia) to 57% (Colombia) of mothers had an acute illness during pregnancy, 2% (Ecuador) to 15% (Argentina) had a chronic illness, and 2% (Bolivia) to 7% (Uruguay) had bleeding during the first trimester. Maternal education differed between countries, with the rate of completing university ranging from under 2% in Venezuela to over 16% in Colombia. Most mothers were unemployed, with rates ranging from 51% (Ecuador) to 90% (Venezuela). For those that were employed the most common occupational activity was either unskilled blue collar or clerk.

**Table 1:** Variable Means and Frequencies in the Analytical Sample by Study Country.

	Brazil	Argentina	Chile	Venezuela	Ecuador	Colombia	Bolivia	Uruguay
N	19,285	12,499	11,617	4,930	2,338	2,232	1,614	1,499
Prenatal care								
Number ofprenatal carevisits (mean,SD)	6.690(2.415)	6.138(2.523)	8.184(1.992)	5.084(2.909)	6.049(2.442)	6.370(2.521)	4.478(2.401)	7.064(2.479)
Birth weight								
Correctedbirth weight(mean (g),SD)	3117.075(595.369)	3267.312(549.667)	3375.228(538.210)	3132.657(513.070)	3054.524(445.396)	2991.223(534.515)	3214.924(503.605)	3237.566(575.085)
Low birthweight (%)	12.32	6.93	5.09	8.68	8.07	14.07	5.55	8.14
Gestationalage in weeks(mean (g),SD)	38.890(3.103)	39.043(2.653)	39.078(2.363)	38.732(2.498)	38.988(2.360)	38.407(3.037)	38.989(2.610)	39.241(2.731)
Sex ofinfants (%)								
Male	54.43	53.48	54.48	54.75	55.18	55.15	53.22	57.17
Female	45.47	46.52	45.52	45.25	44.82	44.85	46.78	42.83
Ancestry (%)								
Other	18.33	15.20	7.43	0.43	40.85	3.05	0.25	58.71
Native	25.85	84.44	92.40	80.28	55.86	93.95	99.32	34.76
African	55.82	0.36	0.17	19.29	3.29	3.00	0.43	6.54
Maternalhealth (%)								
Acute illnessduringpregnancy	47.36	36.14	37.01	31.91	33.62	57.03	23.85	29.29
Chronicillness duringpregnancy	15.01	15.14	14.63	3.89	2.14	11.02	4.34	12.61
Bleeding infirst trimester	5.36	5.02	4.14	1.83	2.82	3.94	1.86	7.14
Fertilityhistory								
Conceptiondifficulty (%)	9.19	7.66	5.54	1.76	2.35	3.27	2.97	11.27
Number ofprevious livebirths (mean,SD)	1.415(1.911)	2.038(2.488)	1.370(1.749)	1.907(2.382)	1.362(2.001)	1.138(1.526)	1.657(2.248)	1.302(1.835)
Number ofspontaneousabortions orstill births(mean, SD)	0.274(0.788)	0.269(0.723)	0.239(0.735)	0.244(0.671)	0.169(0.606)	0.240(0.697)	0.207(0.699)	0.282(0.799)
Maternal age(%)								
Ages 20–25	34.25	36.56	30.52	37.46	37.47	31.81	37.61	28.82
Ages 13–19	23.08	20.47	20.09	28.24	19.33	23.30	17.84	12.27
Ages 26–34	32.08	32.55	35.69	27.83	33.62	33.11	32.47	43.96
Ages 35–49	10.59	10.42	13.70	6.47	9.58	11.78	12.08	14.94
Maternaleducation (%)								
Did notattend orcompleteprimaryschool	39.44	13.01	10.8	16.88	8.90	9.86	17.22	4.80
Completedprimaryschool	15.30	32.04	12.94	20.20	22.16	8.74	22.49	20.75
Attended butdid notcompletesecondaryschool	16.55	29.34	28.62	39.05	26.35	25.94	25.22	37.36
Completedsecondaryschool	23.06	17.23	40.54	15.94	22.50	29.03	23.54	15.08
Incompleteuniversity	2.71	5.13	3.39	6.02	10.95	10.13	7.50	10.67
Completeduniversity	2.95	3.22	3.64	1.91	9.15	16.31	4.03	11.34
Maternalemployment (%)								
Unemployed	61.55	80.23	73.64	90.26	51.37	65.77	78.75	55.57
Employed	38.45	19.77	26.36	9.74	48.63	34.23	21.25	44.43
Unskilledblue collar	15.75	6.98	5.81	4.44	11.50	3.76	11.83	3.80
Skilled bluecollar	6.91	2.59	3.33	1.22	6.12	2.33	2.29	2.07
Independent	1.91	1.96	0.82	1.50	2.40	1.66	0.43	1.60
Clerk	11.29	5.52	14.29	2.35	24.08	16.85	5.82	24.28
Professional,executive,boss	2.60	2.72	2.11	0.22	4.49	9.63	0.87	12.68
Paternaleducation(%)								
Did notattend orcompleteprimaryschool	38.25	11.98	9.09	17.42	5.60	7.97	9.79	4.47
Completedprimaryschool	17.13	37.13	11.57	21.16	24.68	10.89	22.18	20.48
Attendedbut did notcompletesecondaryschool	13.40	23.97	26.27	30.00	20.62	19.85	24.91	31.35
Completedsecondaryschool	21.72	17.41	42.92	18.70	27.07	33.11	27.70	18.35
Incompleteuniversity	2.37	3.56	3.54	4.22	10.14	8.47	8.74	8.94
Completeduniversity	2.83	3.15	4.81	3.25	10.78	17.20	5.54	9.27
Missing	4.29	2.80	1.80	5.25	1.11	2.51	1.24	7.14
Paternalemployment (%)								
Unemployed	7.81	10.34	8.81	8.30	4.02	5.91	9.17	5.27
Employed	90.15	87.24	89.80	91.11	94.65	92.21	89.84	87.59
Unskilledblue collar	38.12	28.47	26.28	51.20	26.39	25.81	19.76	18.01
Skilled bluecollar	19.65	23.03	19.10	16.47	14.69	9.86	18.84	8.81
Independent	7.62	13.39	6.61	11.03	8.64	8.69	27.26	6.07
Clerk	19.87	16.66	32.63	10.37	39.05	34.68	22.06	38.36
Professional,executive, boss	4.89	5.70	5.17	2.05	5.90	13.17	1.92	16.34
Missing	2.04	2.42	1.39	0.59	1.33	1.88	0.99	7.14
Paternalcohabitation								
Cohabitationstatus (%)	93.00	90.27	83.43	96.37	93.37	83.60	93.43	91.59
Cohabitationlength (years:mean, SD)	4.624(4.701)	4.771(4.940)	4.238(4.903)	4.129(4.080)	3.510(4.194)	3.657(4.438)	4.503(5.222)	4.603(4.631)

Notes: The Table reports descriptive statistics for the study variables (percentages for categorical variables and means and standard deviations (SD) for continuous variables) using the main analytical sample for each country.

### Association between Prenatal Visits and Fetal Growth

Table 2 shows the prenatal visit coefficients in the OLS regression of BW for each country‘s total analytical sample with complete data on study variables (described in Table 1) and all available birth years. We present three sets of estimates. The first estimates are completely “crude” without adjustment for any covariate. The second specification adjusts for gestational age. The third set of results is from the main specification which adds all the demographic, socioeconomic, maternal, birth year, and hospital controls described above.

**Table 2:** Coefficients and Odds Ratios (ORs) of Prenatal Visits in BW/LBW Regressions.

Country	Unadjusted		Adjusted for Gestational Age Only		Adjusted for All Covariates	
	β(SE) forBW	OR [95%CI] for LBW	β(SE) forBW	OR [95%CI] for LBW	β(SE) forBW	OR [95% CI]for LBW
Brazil	37.90***(3.63)	0.85***[0.83,0.88]	16.60***(1.67)	0.94***[0.92,0.96]	19.85***(2.30)	0.92***[0.90,0.94]
Argentina	30.08***(3.82)	0.85***[0.81,0.89]	12.50***(2.42)	0.96*[0.91,1.00]	14.74***(2.48)	0.95***[0.91,0.99]
Chile	51.16***(7.1)	0.77***(0.04)	23.74***(5.53)	0.91[0.80,1.03]	26.01***(5.36)	0.87***[0.80,0.94]
Venezuela	14.94**(3.83)	0.93***[0.87,0.97]	13.44**(3.02)	0.94***[0.90,0.98]	11.18***(1.85)	0.95***[0.93,0.97]
Ecuador	14.25**(5.04)	0.93**[0.88,0.99]	13.22**(2.79)	0.95[0.88,1.02]	15.26**(5.93)	0.91*[0.82,1.01]
Colombia	28.07***(8.07)	0.86***[0.81,0.92]	15.13**(6.56)	0.92**[0.85,0.996]	21.82***(5.19)	0.91***[0.89,0.94]
Bolivia	24.03***(4.78)	0.79***[0.76,0.81]	13.94**(4.03)	0.84***[0.81,0.88]	9.06***(1.68)	0.89***[0.86,0.92]
Uruguay	63.15***(1.94)	0.78***(0.73,0.84]	41.0***(1.09)	0.87***[0.84,0.90]	35.64***(2.45)	0.89***[0.87,0.91]

Notes: *, **, and *** indicate p<0.1, p<0.05, and p<0.01 respectively. The sample sizes for OLS regressions for BW are the same as those listed in Table 1. Some covariates (e.g. certain hospital fixed effects) with very few observation frequencies that predicted LBW perfectly were automatically dropped with their observations from the logistic regression model to improve model convergence and fit, resulting in a slightly smaller sample size for the logistic regression than the OLS in all countries except Chile.

Prenatal care was positively and significantly (at least at p<.05) associated with BW for all countries. For all countries, the “crude” estimates without adjustment for gestational age or other covariates were the largest as expected, ranging from 14 grams per visit for Ecuador to 63 grams for Uruguay. Also as expected, adjusting for gestational age reduced the prenatal care coefficients, which now ranged from 13 grams per visit for Argentina to 41 grams for Uruguay. For most countries, adjusting for the additional household, birth year, and hospital effects slightly increased the prenatal care coefficient, suggesting a negative bias of adverse self-selection into prenatal care (i.e. women at greater risk for lower BW obtaining more visits) when excluding these variables. In contrast, the prenatal care coefficient decreased with this adjustment for three countries – Venezuela, Bolivia and Ecuador – suggesting the opposite bias from excluding these covariates. In the most adjusted and preferred specification, the OLS coefficient remained highest for Uruguay at 36 grams per visit and was lowest for Bolivia at 9 grams per visit. Chile had the second largest coefficient (26 grams per visit) followed by Colombia (22 grams), Brazil (20 grams), Ecuador and Argentina (15 grams), and Venezuela (11 grams).

Also included in Table 2 are the odds ratios (ORs) for prenatal visits from the logistic regression of LBW based on the total analytical sample for each country. We present the results from the three specifications described above. The pattern of changes in results with adjusting was overall similar to that for BW. However, the ranking of the countries on the strength of association with LBW was not identical to that of BW. This is not surprising given that the magnitude of association between prenatal care and BW has been shown to vary at different percentiles of the BW distribution with particularly larger association at the left margin of this distribution where LBW children are [14]. Therefore, differences between countries in the magnitude of association between prenatal care and BW mean (estimated from OLS) may vary from that for LBW. In the most adjusted specification, the association with LBW was strongest for Chile (OR = 0.87) and lowest for Argentina and Venezuela (OR = 0.95); the other countries had ORs close to 0.9.

As mentioned above (section 2.1), we evaluated the association between prenatal visits and fetal growth excluding mothers who had 9 or more visits to avoid the data entry limitation of capping 9 or more visits at 9 in the earlier years of data collection. The goal was to ensure that the observed associations described above are not extensively affected by this limitation. The results are reported in Table 3 only for the most adjusted specification. For most countries, this exclusion had little effect on the results except for Ecuador, where the coefficient in the OLS regression for BW dropped by more than half and became insignificant (the association with LBW in Ecuador also became insignificant). The OLS coefficient for Uruguay dropped from 36 to 23 grams per visit but the association for LBW remained the same. These results suggest that this data limitation had overall no effect on our findings for most countries except for the results for Ecuador which should be interpreted more cautiously.

**Table 3:** Coefficients and Odds Ratios (ORs) of Prenatal Visits in BW/LBW Regressions Excluding Mothers at ≥9 Visits.

Country	Coefficient (SE) fromOLS regression for BW	OR [95% CI] fromlogistic regression for LBW	N
Brazil	18.61 *** (2.78)	0.93*** [0.91,0.94]	13,943
Argentina	17.40*** (2.48)	0.94** [0.89,0.99]	9,413
Chile	25.14*** (5.98)	0.94 [0.86,1.02]	4,582
Venezuela	12.27*** (1.77)	0.95*** [0.91,0.98]	4,269
Ecuador	6.08 (6.8)	0.99 [0.88, 1.10]	1,980
Colombia	25.26*** (4.0)	0.87*** [0.83,0.91]	1,767
Bolivia	10.78*** (1.46)	0.92*** [0.90,0.94]	1,549
Uruguay	22.56*** (5.21)	0.89*** [0.83,0.96]	816

Notes: ** and *** indicate p<0.05 and p<0.01, respectively. The sample sizes (N) for the OLS regressions for BW are reported. Some covariates (e.g. certain hospital fixed effects) with very few observation frequencies that predicted LBW perfectly were automatically dropped with their observations from the logistic regression model to improve model convergence and fit, resulting in a slightly smaller sample size for the logistic regression than the OLS in all countries except Chile.

Next, we show in Table 4 the results for two time periods, 1996–2002 and 2003–2011, for the five countries with data on the entire period (1996–2011). As mentioned above, this analysis is limited to mothers who had 8 or fewer prenatal visits (including no visits) to avoid the data limitation of capping 9 or more visits at 9 in the earlier years. The association between prenatal visits and BW/LBW decreased noticeably in the second period in all countries except for the association with BW in Argentina which slightly increased. In the earlier period, prenatal visits were significantly associated with an increase in BW mean in all countries – from 13 grams per visit in Venezuela to 39 grams per visit in Chile. In contrast, this association was lower in the second period with a range from 7 grams per visit in Bolivia to 18 grams per visit in Argentina; the association declined to 10 grams per visit in Chile and became insignificant. Similarly, the association between prenatal visits and LBW became smaller for all countries in the second period and insignificant except for Brazil.

**Table 4:** Coefficients and Odds Ratios (ORs) of Prenatal Visits in BW/LBW Regressions Stratifying by Child’s Birth Period.

Country	Born in 1996–2002			Born in 2003–2011		
	N	Coefficient (SE) fromOLS regression forBW	OR [95% CI] fromlogistic regressionfor LBW	N	Coefficient (SE) fromOLS regression for BW	OR [95% CI] fromlogistic regressionfor LBW
Brazil	6,331	23.19*** (4.67)	0.89*** [0.86, 0.92]	7,612	14.19*** (3.39)	0.96*** [0.94,0.99]
Argentina	5,589	16.31*** (2.85)	0.92*** [0.87, 0.97]	3,824	18.43*** (4.72)	0.97 [0.91,1.04]
Chile	2,485	39.32*** (6.33)	0.90*** [0.83,0.97]	2,097	9.96 (6.94)	0.96 [0.86,1.08]
Venezuela	2,460	13.28*** (0.81)	0.94** [0.90,0.99]	1,809	9.04** (2.47)	0.93 [0.86,1.02]
Bolivia	644	17.78** (2.19)	0.92*** [0.91,0.92]	905	6.89** (2.16)	0.93 [0.85,1.02]

Notes: *** and ** indicate p<0.01 and p<0.05 respectively.

Mothers with 9 or more prenatal visits are excluded from the analysis. The sample sizes (N) for the OLS regressions for BW are reported. Some covariates (e.g. certain hospital fixed effects) with very few observation frequencies that predicted LBW perfectly were automatically dropped with their observations from the logistic regression model to improve model convergence and fit, resulting in a slightly smaller sample size for the logistic regression than the OLS in all countries.

## Discussion

This study is one of the first to examine and compare the association between prenatal care utilization and fetal growth measured by birth weight adjusted for gestational age across several South American countries using similarly collected data and the same analytical model across all countries. We found positive associations in all countries, consistent with previous research [3], [11], [13], [26], [27]. However, there were clear differences in the magnitude of this association between the eight studied countries, by up to 27 grams per visit for BW and 8% lower odds of LBW per visit based on the most adjusted specification. Our study can only estimate associations and these estimates should not be interpreted as causal effects. However, these differences suggest that as one of the commonly used indicators for a country’s healthcare system for maternal and child health, prenatal care is a highly variable indicator in terms of its implication for fetal growth between countries in South America, including neighbors that may be thought to be relatively similar in their population demographics, economics, and even health care systems. Our study suggests that Uruguay and Chile had distinctively larger associations between prenatal care and fetal growth than the other countries over the last two decades, while Venezuela had the lowest association. One implication of these results is that estimates of association between prenatal care and BW are population-specific and may not be generalizable to other populations. Recognizing this heterogeneity is important to avoid potentially inapplicable generalization of results between countries.

Explaining the observed between-country differences in prenatal care associations is outside the scope of the current study, but some of the differences between the country samples may offer some clues about population factors that may modify these associations. For example, the rates of chronic illnesses during pregnancy and conception difficulty were lowest in Venezuela and Ecuador (Table 1) where prenatal care was least associated with fetal growth. Previous work has shown that prenatal care is more relevant for pregnancies at greater risks of having lower BW infants [14]. Explaining these country differences requires additional datasets and models but is an important topic for future research.

Our analysis of changes in magnitude of prenatal care associations with fetal growth over time indicated that overall this association has decreased over the last decade. Explaining this change requires separate work and modeling in future research but some potential pathways are worth discussing. Increased access to prenatal care over time, including for women with low-risk pregnancies for whom prenatal care may be associated with a lower increase in BW than those with more complicated pregnancies may play a role. Other factors may be changes in population health indicators that are targeted by prenatal care, such as maternal smoking, which have been decreasing over time, and maternal nutrition, which have generally improved over time. Some of the variation in prenatal care associations over time may also be driven by changes in the content and quality of prenatal care practices, and the extent to which care is focused on technology such as ultrasound evaluations versus direct interactions between health professionals and patients that can identify and address risks including health behaviors. The current paper does not evaluate predictors of prenatal care use or quality and therefore cannot offer specific insights about how prenatal care content and quality can be improved. We believe this an important question for future research where prenatal care use is analyzed as the outcome.

Our study has several strengths including a large and socioeconomically and demographically diverse sample for each country (as shown in Table 1), evaluating multiple countries with similar data and methods, and rich data on multiple risk factors and confounders. A power analysis showed high power in all of the total country samples (∼1 for Brazil, Argentina, Chile, Colombia, and Uruguay, and 0.98 for Venezuela and Ecuador) except for Bolivia (power of 0.39) to detect the observed association between prenatal care and BW based on the OLS regression adjusting for all covariates. However, there are also limitations that warrant discussion. One limitation is the possibility of confounding due to unobservable factors related to both maternal self-selection into prenatal care and fetal growth such as the mother’s preferences for her and her child’s health and her health behaviors and conditions. In a sensitivity analysis, we added an indicator for maternal hypertension during pregnancy as a covariate given that it is a well-recognized risk factor for fetal growth retardation. Data on this condition was inadequately captured in Ecuador and Bolivia so these countries were excluded from this analysis. Adding this variable had no effect on the coefficients of prenatal care. Hypertension was significantly related to BW/LBW in all 6 analyzed countries except Colombia, with nearly doubled odds of LBW in Brazil, Argentina, Chile and Venezuela, a five-fold increase in LBW odds in Uruguay, and decrease in BW mean of 84 and 129 grams in Brazil and Chile. Nonetheless, as stated above, we are only able to estimate associations and not causal effects. A few previous studies in South American populations using designs that can account for unobservable confounders including instrumental variables have generally found evidence of adverse self-selection (on unobservable characteristics) into prenatal care, with mothers at greater risk for having lower BW infants seeking more prenatal care [14], [15], [20]. If so, our estimates may be a lower bound of the real relationship between prenatal care and BW. We do not have access to data that allow for applying methods such as instrumental variables that may identify causal effects in observational data as used in these studies [14], [15].

Another limitation is the capping of visits above 9 at data entry in earlier years of data collection as explained above. However, this had little effect for all countries except Ecuador (Table 2). For Ecuador, the estimate was sensitive to excluding mothers who had 9 or more visits, even though the majority of mothers (about 85%) obtaining prenatal care had fewer than 9 visits. This suggests that the association with birth weight in this country was mostly driven by women who obtained 9 or more visits. This can due to differences in the characteristics of these women from those who obtained fewer visits or due to a difference in prenatal care content. A comparison of the women who obtained 9 or more visits in Ecuador with those who obtained fewer or no visits revealed that the first group had more health risks associated with lower BW including higher rates of acute and chronic illnesses, vaginal bleeding during pregnancy, and conception difficulty, fewer previous live births, and more previous miscarriages/stillbirths (details available upon request). As mentioned above, previous work is suggestive of prenatal care being more strongly associated with birth weight for more complicated pregnancies [14]. Also, women who received 9 or more visits were more educated on average (27% attended/completed university compared to 18%), which may further allow them to benefit more from prenatal care. However, the sensitivity of the estimates to excluding the small subset with 9 or more visits calls for caution in interpreting the results for Ecuador.

Another limitation is that the samples may not be fully representative of the infant population in each country given that these are obtained from selective hospitals. This could limit the generalizability of the results for each country and bias the estimated differences between the country samples compared to population differences. The extensive variation in the demographic, socioeconomic, and maternal health characteristics and the multiple geographic regions covered within each country sample suggests that these samples are highly diverse and reflect multiple backgrounds within each country. Therefore, while they may not be fully representative of the entire country populations, these samples are likely representative of an important proportion of these populations. We are unable to fully examine the representativeness of the samples as most of the study countries only provide data on a few variables for their birth population. When comparing the LBW rates in our samples to those from “national” estimates for the study countries, we find that they are within range of estimates reported for Argentina, Chile, Venezuela, and Uruguay, relatively close to reported range for Brazil, Ecuador, and Bolivia, and noticeably out of range for Colombia (see supplementary Table S2 online). The extensive within-country geographic differences in LBW rate in some countries such as in Brazil may explain why our samples may be slightly out of range for such countries [28]. The LBW rate has also been reported to vary widely within Colombia, with a rate of over 12% in Bogota compared to a national rate range of 6–9% [29], possibly due to the greater LBW risk with altitude [30]. Nearly two-thirds of our sample from Colombia was born above 2,000 meters, which may explain the higher than national LBW rate.

Similar to most previous studies of prenatal care, our results are applicable to live births as we have no prenatal care and BW data for miscarriages and stillbirths. Three other aspects related to the representativeness of our sample are worth discussing. One issue is the overrepresentation of males (54% males, 46% females) which is due to the higher prevalence of certain common birth defects such as oral clefts among males and the one-to-one matching of infants without birth defects to those with birth defects based on gender in ECLAMC. Even though we only include infants without birth defects in our analysis, this matching results in overrepresentation of males in the dataset. To evaluate if the association between prenatal care and birth weight varies by gender, we re-estimated the regression models including an interaction term between prenatal visits and gender and found small and insignificant interactions for all countries except in Argentina and Colombia, where the association between prenatal visits and BW was stronger for males than females. In Argentina, BW increased by 20 (8) grams per visit for males (females). In Colombia, BW increased by 30 (10) grams per visit for males (females). This heterogeneity by gender could reduce the representativeness of the results to the total infant populations in these two countries. Another issue is that our sample only includes in-hospital births, making the results applicable only to infants born in healthcare institutions (hospitals). However, this is a minor limitation since the majority of infants are born in healthcare institutions in the study populations. Furthermore, we are unable to compare our sample to infants born outside of the hospital in the study communities/countries as data are unavailable for them.

The third issue related to sample representativeness is that about one third of the total sample was excluded from the final analytical sample, mostly due to missing data (see Figure 1). We do not impose any inclusion/exclusion criteria on the analytical sample other than complete and sensible data on the study variables and have no reason to believe a-priori that data are missing non-randomly on BW, prenatal care visits, or other major variables. However, we cannot rule out this possibility which may also affect generalizability. In order to further evaluate potential sample selection bias from missing data, we estimated a basic regression of BW on prenatal visits adjusting only for hospital and year dummies first for observations with complete data on birth weight and prenatal visits (regardless of data completion on other covariates included in the main model); the total group included 76,002 observations. Next, we estimated this same basic regression for observations with complete data on all variables (including the covariates adjusted for in the main model); this group included the analytical samples used in the main models which totaled 56,104 observations. The prenatal care coefficients were very close between the two groups for all countries (within 3 grams per visit) except for Chile where the coefficients were different by about 9 grams per visit (50 grams in the larger group including observations with missing data on additional covariates versus 59 grams in the analytical sample with complete data). These results provide some assurance that missing data did not occur systematically and did not substantially bias the association between prenatal care and BW in the analytical samples with complete data on covariates.

To the best of our knowledge, ECLAMC is one of the few resources (if not the only resource) with detailed infant-level data collected using the same methods across all study countries for such a comparative analysis. As national datasets with similar design and data become available for the study countries, replicating this study using these resources would be an important future research direction.

## Supporting Information

Table S1 **Number of observations with complete data for each variable and percentage out of sample with data on BW and prenatal care.**
(DOCX)

Table S2 **“National” Estimates of LBW Rates in Study Countries Compared to our Study Sample Estimates.**
(DOCX)

## References

[1] Cox RG, Zhang L, Zotti ME, Graham J (2011) Prenatal care utilization in Mississippi: racial disparities and implications for unfavorable birth outcomes. Matern Child Health J 15: 931–942. 19943096 10.1007/s10995-009-0542-6

[2] Zhang L, Cox RG, Graham J, Johnson D (2011) Association of maternal medical conditions and unfavorable birth outcomes: findings from the 1996–2003 Mississippi linked birth and death data. Matern Child Health J 15: 910–920. 19760166 10.1007/s10995-009-0516-8

[3] Carvalho Padilha PD, Accioly E, Chagas C, Portela E, Da Silva CL, et al (2009) Birth weight variation according to maternal characteristics and gestational weight gain in Brazilian women. Nutr Hosp 24: 207–212. 19593493

[4] Ferri CP, Mitsuhiro SS, Barros MC, Chalem E, Guinsburg R, et al (2007) The impact of maternal experience of violence and common mental disorders on neonatal outcomes: a survey of adolescent mothers in Sao Paulo, Brazil. BMC Public Health 7: 209. 17705835 10.1186/1471-2458-7-209 PMC2018717

[5] VanderWeele TJ, Lantos JD, Siddique J, Lauderdale DS (2009) A comparison of four prenatal care indices in birth outcome models: comparable results for predicting small-for-gestational-age outcome but different results for preterm birth or infant mortality. J Clin Epidemiol 62: 438–445. 18945589 10.1016/j.jclinepi.2008.08.001

[6] WHO (2012) Maternal, newborn, child and adolescent health: care of the preterm and/or low-birth-weight newborn.: World Health Organization.

[7] Martin JA, Hamilton BE, Ventura SJ, Osterman MJ, Kirmeyer S, et al (2011) Births: final data for 2009. Natl Vital Stat Rep 60: 1–70. 22670489

[8] Varvarigou AA (2010) Intrauterine growth restriction as a potential risk factor for disease onset in adulthood. J Pediatr Endocrinol Metab 23: 215–224. 20480719 10.1515/jpem.2010.23.3.215

[9] Lau C, Rogers JM, Desai M, Ross MG (2011) Fetal programming of adult disease: implications for prenatal care. Obstet Gynecol 117: 978–985. 21422872 10.1097/AOG.0b013e318212140e

[10] WHO (2012) World health statistics 2012. Geneva: WHO Department of Health Statistics and Information Systems.

[11] You D, New JR, Wardlaw T (2012) Levels & Trends in Child Mortality, Report 2012. New York: UNICEF, WHO, World Bank, United Nations.

[12] Wardlaw T, Blanc A, Zupan J, Ahman E (2004) Low birthweight: country, regional, and global estimates. New York: UNICEF 31 p.

[13] Wehby GL, Murray JC, Castilla EE, Lopez-Camelo JS, Ohsfeldt RL (2009) Prenatal care effectiveness and utilization in Brazil. Health Policy Plan 24: 175–188. 19282483 10.1093/heapol/czp005 PMC2708921

[14] Wehby GL, Murray JC, Castilla EE, Lopez-Camelo JS, Ohsfeldt RL (2009) Quantile effects of prenatal care utilization on birth weight in Argentina. Health Econ 18: 1307–1321. 19142894 10.1002/hec.1431 PMC2763933

[15] Wehby GL, Murray JC, Castilla EE, Lopez-Camelo JS, Ohsfeldt RL (2009) Prenatal care demand and its effects on birth outcomes by birth defect status in Argentina. Econ Hum Biol 7: 84–95. 19059012 10.1016/j.ehb.2008.10.001 PMC2680816

[16] Donaldson PJ, Billy JO (1984) The impact of prenatal care on birth weight. Evidence from an international data set. Med Care 22: 177–188. 6700278 10.1097/00005650-198402000-00009

[17] Jewell RT, Triunfo P (2006) The impact of prenatal care on birthweight: the case of Uruguay. Health Econ 15: 1245–1250. 16786548 10.1002/hec.1121

[18] Liu X, Zhang J, Liu Y, Li Y, Li Z (2012) The association between cesarean delivery on maternal request and method of newborn feeding in China. PLoS One 7: e37336. 22624019 10.1371/journal.pone.0037336 PMC3356247

[19] WBG (2012) World dataBank. USA: The World Bank Group.

[20] Jewell RT (2007) Prenatal care and birthweight production: evidence from South America. Applied Economics 39: 415–426.

[21] Castilla EE, Orioli IM (2004) ECLAMC: The Latin-American Collaborative Study of Congenital Malformations. Community Genetics 7: 76–94. 15539822 10.1159/000080776

[22] Nyarko KA, Lopez-Camelo J, Castilla EE, Wehby GL (2013) Does the Relationship between Prenatal Care and Birth Weight Vary by Oral Clefts? Evidence Using South American and United States Samples. J Pediatr 162: 42–49 e41. 10.1016/j.jpeds.2012.06.040 PMC3485451 22835882

[23] Wehby GL, Nyarko KA, Lopez-Camelo JS (2014) Fetal Health Shocks and Early Inequalities in Health Capital Accumulation. Health Econ 23: 69–92. 23339079 10.1002/hec.2901 PMC3865137

[24] Serruya SJ, Cecatti JG, Lago T (2004) [The Brazilian Ministry of Health’s Program for Humanization of Prenatal and Childbirth Care: preliminary results]. Cad Saude Publica 20: 1281–1289. 15486671 10.1590/s0102-311x2004000500022

[25] Wooldridge JM (2002) Econometric Analysis of Cross Section and Panel Data. Cambridge, MA and London, England: MIT Press.

[26] Reichman NE, Corman H, Noonan K, Dave D (2009) Infant health production functions: what a difference the data make. Health Econ 18: 761–782. 18792077 10.1002/hec.1402 PMC2697267

[27] Abrevaya J (2001) The effects of demographics and maternal behavior on the distribution of birth. Empirical Economics 26: 247–257.

[28] Nyarko KA, Lopez-Camelo J, Castilla EE, Wehby GL (2013) Explaining Racial Disparities in Infant Health in Brazil. American Journal of Public Health 103: 1675–1684. 23409894 10.2105/AJPH.2012.301021 PMC3740020

[29] WHO (2013) Columbia. Pan-American Health Organization. www.paho.org/saludenlasamericas/index.php?id=30&option=com_content&ltemid=&lang=pt#ref17.

[30] Wehby GL, Castilla EE, Lopez-Camelo J (2010) The impact of altitude on infant health in South America. Economics & Human Biology 8: 197–211. 20594925 10.1016/j.ehb.2010.04.002 PMC2914839

