# Supplementary material for: A Comparative Analysis of Prenatal Care and Fetal Growth in Eight South American Countries
Source: PLoS One. 2014 Mar 13;9(3):e91292. doi: 10.1371/journal.pone.0091292 (PMC3953331; doi:10.1371/journal.pone.0091292)
Supplement: Table S1 — Number of observations with complete data for each variable and percentage out of sample with data on BW and prenatal care. (DOCX) [file pone.0091292.s001.docx]

**Supplementary Table S1. Number of observations with complete data for each variable and percentage out of sample with data on BW and prenatal care**

| Variable | Brazil | Argentina | Chile | Venezuela | Colombia | Ecuador | Bolivia | Uruguay |
| --- | --- | --- | --- | --- | --- | --- | --- | --- |
| Sample with data on BW and prenatal care | 26,217 | 16,908 | 14,334 | 5,763 | 3,332 | 3,197 | 2,068 | 2,096 |
| Gestational Age | 23,475 (89.54%) | 14,969  (88.53%) | 13,916  (97.08%) | 5,386  (93.46) | 2,960  (88.84%) | 2,992  (93.59%) | 1,992  (96.32) | 1,835  (87.55%) |
| Maternal age | 26,158  (99.77%) | 16,899  (99.95%) | 14,304  (99.79%) | 5,760  (99.95%) | 3,323  (99.73%) | 3,196  (99.97%) | 2,066  (99.90%) | 2,093  (99.86%) |
| Female/male | 26,212  (99.98%) | 16,906  (99.99%) | 14,334  (100%) | 5,763  (100%) | 3,331  (99.97%) | 3,196  (99.97%) | 2,068  (100%) | 2,096  (100%) |
| Race | 25,383  (96.82%) | 16,688  (98.70%) | 14,172  (98.87%) | 5,737  (99.55%) | 3,251  (97.57%) | 3,171  (99.19%) | 2,059  (99.56%) | 2,066  (98.57%) |
| Vaginal bleeding in first trimester | 25,308  (96.53%) | 16,393  (96.95%) | 13,934  (97.21%) | 5,657  (98.16%) | 3,164  (94.96%) | 3,036  (94.96%) | 1,976  (95.55%) | 2,067  (98.62%) |
| Number of still births or spontaneous abortions | 24,049  (91.73%) | 15,744  (93.12%) | 13,288  (92.71%) | 5,533  (96.01%) | 2,974  (89.26%) | 2,769  (86.61%) | 1,952  (94.39%) | 2,080  (99.24%) |
| Acute maternal illness during pregnancy | 26,140  (99.71) | 16,810  (99.42%) | 14,257  (99.46%) | 5,759  (99.93%) | 3,325  (99.79%) | 3,181  (99.50%) | 2,048  (99.03%) | 2,065  (98.52%) |
| Chronic maternal illness during pregnancy | 26,124  (99.65%) | 16,816  (99.46%) | 14,271  (99.56%) | 5,749  (99.76%) | 3,325  (99.79%) | 3,170  (99.16%) | 2,009  (97.15%) | 2,071  (98.81%) |
| Number of previous live births | 24,049  (91.73%) | 15,744  (93.12%) | 13,288  (92.71%) | 5,533  (96.01) | 2,974  (89.26%) | 2,769  (86.61%) | 1,952  (94.39%) | 2,080  (99.24%) |
| Difficulty with conception | 25,702  (98.04%) | 16,612  (98.25%) | 14,130  (98.58%) | 5,673  (98.44%) | 3,242  (97.30%) | 3,159  (98.81%) | 2,028  (98.07%) | 1,983  (94.61%) |
| Mother’s education level | 26,070  (99.44%) | 16,820  (99.48%) | 14,303  (99.78%) | 5,739  (99.58%) | 3,320  (99.64%) | 3,174  (99.28%) | 2,061  (99.66%) | 2,085  (99.48%) |
| Mother’s employment | 25,942  (98.95%) | 16,727  (98.93%) | 14,222  (99.22%) | 5,730  (99.43%) | 3,296  (98.92%) | 3,171  (99.19%) | 2,054  (99.32%) | 2,048  (97.71%) |
| Length of parental cohabitation | 25,428  (96.99%) | 16,039  (94.86%) | 13,832  (96.50%) | 5,686  (98.66%) | 3,143  (94.33%) | 3,157  (98.75%) | 1,948  (94.20%) | 1,869  (89.17%) |
| Father’s education level | 24,703  (94.23%) | 15,999  (94.62%) | 13,992  (97.61%) | 5,446  (94.50%) | 3,174  (95.26%) | 3,135  (98.06%) | 2,022  (97.78%) | 1,830  (87.31%) |
| Father’s employment | 25,361  (96.73%) | 16,070  (95.04%) | 14,032  (97.89%) | 5,697  (98.95%) | 3,202  (96.10%) | 3,132  (97.97%) | 2,027  (98.02%) | 1,831  (87.36%) |
