# Supplementary material for: A Comparative Analysis of Prenatal Care and Fetal Growth in Eight South American Countries
Source: PLoS One. 2014 Mar 13;9(3):e91292. doi: 10.1371/journal.pone.0091292 (PMC3953331; doi:10.1371/journal.pone.0091292)
Supplement: Table S2 — “National” Estimates of LBW Rates in Study Countries Compared to our Study Sample Estimates. (DOCX) [file pone.0091292.s002.docx]

**Table S2. “National” Estimates of LBW Rates in Study Countries Compared to our Study Sample Estimates**

| **Data Source** | **Our Data** | **UNICEF (1)** | **UNICEF (2)** | **WHO (3)** | **WHO (4)** | **UN (5)** |
| --- | --- | --- | --- | --- | --- | --- |
| ***Years*** | ***1996-2011*** | ***1996-2001*** | ***1999-2009*** | ***2008-2009*** | ***All 2005-2010*** | ***All 2007-2011*** |
| **Brazil** | 12.32% | 1996: 10% | 2005: 8% | 2009: 8% | 8% | 8% |
| **Argentina** | 6.93% | 1999: 7% | 2006: 7% | 2009: 7% | 7% | 7% |
| **Chile** | 5.09% | 2001: 5% | 2005: 6% | 2008: 6% | 6% | 6% |
| **Venezuela** | 8.68% | 2000: 7% | 2006: 9% | No data | 8% | 8% |
| **Ecuador** | 8.07% | 1999: 16% | 1999: 16% | No data | 8% | 10% |
| **Colombia** | 14.07% | 2000: 9% | 2000: 9% | No data | 6% | 6% |
| **Bolivia** | 5.55% | 1996: 9% | 2003: 7% | No data | 6% | 6% |
| **Uruguay** | 8.14% | 2002: 8% | 2009: 9% | 2009:9% | 9% | 9% |

Notes: The Table reports the LBW rates by country from our analytical sample and “national” estimates reported for the study countries from various sources and periods. When the national estimate was reported for a specific year, that year is included before the estimate.

References:

1. Low birthweight: Country, regional, and global estimates.  World Health Organization and UNICEF. 2004.  Accessed from: <http://www.who.int/reproductivehealth/publications/monitoring/9280638327/en/>
2. Child info: Monitoring the situation of children and women. UNICEF.  2013 Feb.  Accessed from:   <http://www.childinfo.org/low_birthweight_table.php>
3. Global Health Observatory: Country statistics.  World Health Organization. Accessed 2014 Jan from:  <http://www.who.int/gho/countries/en/index.html>
4. World Health Statistics 2012. World Health Organization.  2012.  Accessed from:

<http://apps.who.int/iris/bitstream/10665/44844/1/9789241564441_eng.pdf?ua=1>

1. Proportion of infants with low birth weight.  UN Data.  Accessed 2014 Jan from: http://data.un.org/Data.aspx?d=SOWC&f=inID%3A115
